# Immunity-Related Protein Expression and Pathological Lung Damage in Mice Poststimulation with Ambient Particulate Matter from Live Bird Markets

**DOI:** 10.3389/fimmu.2016.00252

**Published:** 2016-06-27

**Authors:** Kai Meng, Bo Wu, Jing Gao, Yumei Cai, Meiling Yao, Liangmeng Wei, Tongjie Chai

**Affiliations:** ^1^Sino-German Cooperative Research Centre for Zoonosis of Animal Origin Shandong Province, College of Veterinary Medicine, Shandong Agricultural University, Tai’an, China; ^2^Collaborative Innovation Centre for the Origin and Control of Emerging Infectious Diseases, Taishan Medical College, Tai’an, China; ^3^Taian Central Hospital, Tai’an, China; ^4^Zaozhuang Vocational College, Shandong, China

**Keywords:** particulate matter, biological material, live bird market, immunity-related protein, lung injury

## Abstract

The objective of this study was to obtain insight into the adverse health effects of airborne particulate matter (PM) collected from live bird markets and to determine whether biological material in PM accounts for immune-related inflammatory response. Mice were exposed to a single or repeated dose of PM, after which the expression of toll-like receptors (TLRs), cytokines, and chemokines in the lungs of infected mice were examined by enzyme-linked immunosorbent assay and histopathological analysis. Results after single and repeated PM stimulation with ${\text{PM}}_{2.5}^{+},{\text{PM}}_{2.5}^{-},{\text{PM}}_{10}^{+},{\text{ and PM}}_{10}^{-}$ indicated that TLR2 and TLR4 played a dominant role in the inflammatory responses of the lung. Further analysis demonstrated that the expression levels of IL-1β, TNF-α, IFN-γ, IL-8, IP-10, and MCP-1 increased significantly, which could eventually contribute to lung injury. Moreover, biological components in PM were critical in mediating immune-related inflammatory responses and should therefore not be overlooked.

## Introduction

Particulate matter (PM) is a mixture of airborne particles originating from the breakdown of crustal components or from the combustion of fossil fuels. Air pollution caused by ambient PM is a growing problem not only in developing countries but also in the developed world and has resulted in severe hazards for public health (1). Numerous epidemiological studies have indicated that exposure to respirable PM is critical in the exacerbation of chronic obstructive pulmonary disease and asthma as well as it is strongly associated with cardiopulmonary mortality and morbidity in exposed populations, especially in susceptible ones (2). According to the US Environmental Protection Agency, particle pollution includes inhalable coarse particles between 10 and 2.5 μm in diameter (PM_10_) and fine particles less than 2.5 μm in diameter (PM_2.5_). Numerous studies have suggested that fine particles (PM_2.5_) are more closely associated with both acute and chronic respiratory effects, as well as subsequent mortality, than coarse particles (PM_10_) due to their smaller size, larger surface area, deeper airway penetration, and greater ability to be retained in the lungs (3). Although epidemiological reports on adverse health effects are mostly consistent in their views on PM exposure, only a few pay attention to the source and composition of particles that might be responsible for those adverse effects (4). At the same time, the exact constituents of airborne PM that cause airway inflammatory response and their precise mechanisms have yet to be thoroughly investigated (5, 6).

The majority of the Chinese population eats fresh birds (e.g., chickens, ducks, geese, pigeons, and migratory birds). To meet those dietary demands, live bird markets (LBMs) have been established in nearly all cities in China, mostly in densely populated areas. The airborne elements of LBMs contain several harmful biological components (e.g., Gram-negative bacteria, fungi, endotoxins, and influenza virus), and in recent years, the emergence of various highly pathogenic avian influenza viruses (AIVs), including H5N1 (7), H7N9 (8), and H10N8 (9), in China has presented a serious threat to people’s health. It has been reported that these highly pathogenic AIVs can be isolated from LBMs (8, 10–12), and that LBMs are potential hubs for the transmission of AIVs, which can survive there for prolonged periods (13). As early emergency investigations have also shown, LBMs are likely to play an important role in disease transmission (14), which suggests that exposure to airborne PM of LBMs can pose health risks, especially for children, the elderly, and live poultry-slaughtering workers.

Airborne PM contains complex aggregates of inorganic materials (i.e., oxides of transition metals), dust, smoke, metal elements, all kinds of liquid and solid materials in the atmosphere, and biological components, such as bacteria, fungi, and viruses (15, 16). Most studies have examined the role of physical properties (e.g., surface size) or chemical compounds of ambient PM (17, 18), and some researchers have also paid attention to the role of organic compounds (e.g., organic carbon) and polycyclic aromatic hydrocarbons of ambient PM. According to their research, the organic compounds of ambient PM, such as organic carbon and polycyclic aromatic hydrocarbons, can cause severe allergic alveolitis in chickens (19), ducks (20), and even pigeons (21), if exposed to the environment for a long time.

To date, however, the role of biological components has not yet been studied. Airborne PM of LBMs contains numerous biological components, most of which exert adverse health effects on humans, especially LBM customers and workers who slaughter or sell bird products. LBM employees face long-term exposure to such air environments, and airborne PM theoretically affects their respiratory systems. By contrast, customers are exposed to the air environment in the short term, and airborne PM could also affect their respiratory systems. However, to the best of our knowledge, no systematic studies have been conducted on the mechanisms of short- and long-term exposure to LBM environments and its effects on human health, especially that of lung tissue, or the role of biological components in causing pulmonary inflammation.

In response, the purpose of this study was to systematically assess the adverse health effects of airborne PM collected from LBMs on the lungs and to determine whether biological components of airborne PM are essential in mediating the immune-related inflammatory response. Using mice lung tissue, physiological and pathological analyses were also performed to clarify the possible mechanisms that cause inflammation and injury in the lungs.

## Materials and Methods

### Ethics Statement

Animal experiments were reviewed and approved by the Shandong Agriculture University Institutional Animal Care and Use Committee and performed in accordance with the Guidelines for Experimental Animals maintained by China’s Ministry of Science and Technology. The research did not involve endangered or protected species, and animal suffering was minimized to the greatest possible extent.

### Animals

Male BALB/c mice were purchased from Shandong University Laboratory Animal Center. All animal experiments were conducted in a Biosecurity Level 2+ laboratory, with mice kept on a 12-h light–dark cycle in controlled temperature (23–25°C) and humidity (40–60%). Animals were allowed free access to tap water and regular rodent food. Mice that were 6 weeks old and weighing about 13 g at the beginning of the experiments were used. All animals were sacrificed by lethal sodium pentobarbital injection.

### Sample Collection

Atmospheric PM_10_ and PM_2.5_ were collected from an LBM in Tai’an, Shandong Province, China. The sampling location was a semi-open LBM with high population density surrounded by two residential areas, a park, and a vegetable market. The air quality of the LBM appeared to be worse than that of other places and contained more microbial components, especially bacteria, endotoxins, fungi, and even viruses. The PM used was collected in July 2014 with two high-volume air samplers (Thermo Electron Corp., Waltham, MA, USA), one of which was equipped with a PM_2.5_-fractionating inlet and the other with a PM_10_-fractionating inlet. The inlets of the high-volume samplers were roughly 1.5 m above ground level. Ambient air was drawn at a flow rate of 100 L/min for five consecutive days and sampled during July 11–15, 2014. PM (i.e., PM_10_ and PM_2.5_) was collected on 9 cm × 9 cm Tissuquartz™ filters (Pall, Port Washington, NY, USA) with typical aerosol retention of 99.9%. All quartz filters were sterilized by baking in a furnace at 500°C for at least 6 h prior to sampling.

### Sample Handling

We characterized the diversity and relative abundance of bacteria and fungi in airborne PM_2.5_ and PM_10_ by DNA extraction and sequence analysis of 16S RNA and the internal transcribed spacer region, respectively. For the rest of the samples, PM_2.5_ and PM_10_ were divided into two groups – a heat-treated $\left({\text{PM}}_{2.5}^{-}{\text{ and PM}}_{10}^{-}\right)$ and non-heat-treated $\left({\text{PM}}_{2.5}^{+}{\text{ and PM}}_{10}^{+}\right)$ group – after which all particles were again suspended in pyrogen-free phosphate-buffered saline (PBS) in a final concentration of 1 mg/mL. Endotoxin concentrations in fine PM (PM_2.5_ and PM_10_) were assayed in duplicate using a quantitative kinetic chromogenic *Limulus* amebocyte lysate (LAL) method at 37°C. Endotoxin concentrations were expressed as endotoxin units per milligram of fine PM (i.e., EU mg^−1^ PM_2.5_ and EU mg^−1^ PM_10_).

### Study Design

#### Single PM $\left({\text{PM}}_{\text{2.5}}^{+},{\text{PM}}_{\text{2.5}}^{-},{\text{PM}}_{\text{10}}^{+},{\text{ and PM}}_{\text{10}}^{-}\right)$ Stimulation in Mice: Acute Phase Model

Mice were divided into five groups, each for varying PM exposure: the PBS control group; Group A, for heat-treated PM_10_ $\left({\text{PM}}_{10}^{-}\right)$; Group B, for non-heat-treated PM_10_ $\left({\text{PM}}_{10}^{+}\right)$; Group C, for heat-treated PM_2.5_ $\left({\text{PM}}_{2.5}^{-}\right)$; and Group D, for non-heat-treated PM_2.5_ $\left({\text{PM}}_{2.5}^{+}\right)$. For single instillation studies, mice were anesthetized using dry ice, with each mouse grasped in the left hand and its head fully exposed and tilted at about 15°, and 50 μL of the PM suspension (1 mg/mL) was administered to the left nostril of each mouse by micropipette, whereas mice in the control group each received 50 μL of PBS only. Thereafter, the mice were maintained at a constant temperature of 23 ± 2°C and relative humidity of 56 ± 10%, with a 12-h light–dark cycle throughout the study. Each treatment group included six mice, and the experiments were replicated at least three times. Left lung tissue from each group was used for histological analysis, whereas right lung tissue was used to determine the expression of toll-like receptors (TLRs), cytokines, and chemokines after 72 h of PM stimulation.

#### Repeated PM $\left({\text{PM}}_{\text{2.5}}^{+},{\text{PM}}_{\text{2.5}}^{-},{\text{PM}}_{\text{10}}^{+},{\text{ and PM}}_{\text{10}}^{-}\right)$ Stimulation in Mice: Chronic Phase Model

In the chronic phase model, mice from the four PM groups were repeatedly administered PM once per day for five consecutive days per week for four consecutive weeks, under the conditions described above. Body weight was measured twice per week for the entire duration of the experiment. Lungs were extracted 72 h after the final stimulation to determine TLR, cytokine, and chemokine expression and to perform histopathological analysis.

#### Histopathological Evaluation

In single and repeated PM stimulation experiments, mice were sacrificed 72 h after the final administration of PM, and the left lungs of the control and PM-treated mice were excised, immediately fixed with formalin, and processed for routine histology. Briefly, after being preserved for 24 h in 10% neutral-buffered formalin, the lungs were embedded in paraffin, and 4-μm serial sections were cut by rotary microtome and stained with hematoxylin and eosin. Pathological assessments in the bronchus and parenchyma were performed using a light microscope (Eclipse, Nikon, Tokyo, Japan), and lung injury was evaluated by lung injury scores as described in a previous study (22). Briefly, a score of 0 signified no injury, a score of 1 signified injury in 25% of the lung, a score of 2 signified injury in 50% of the lung, a score of 3 signified injury in 75% of the lung, and a score of 4 signified injury throughout the lung. Eight random microscopic fields from each slide were analyzed, and their average score was used to assess the severity of lung injury.

#### Enzyme-linked Immunosorbent Assay to Quantify Immunity-Related Protein Levels

Right lung tissues of control and PM-treated mice were homogenized to 2% in PBS with 0.05% Tween-20 (pH 7.4) using a tissue homogenizer. The resultant homogenate was centrifuged (5,000 × *g* for 10 min, 4°C) and the supernatant placed into sterile microcentrifuge tubes (Eppendorf, Hamburg, Germany). Total protein concentrations in the supernatant of the five groups were adjusted to the same level before measurement. The protein levels of TLRs (i.e., TLR2, TLR3, TLR4, TLR7, TLR8, and TLR9), cytokines (i.e., IFN-α, IFN-β, IFN-γ, TNF-α, IL-1β, IL-6, and IL-10), and chemokines (i.e., IL-8, MCP-1, IP-10, RANTES, and MIP-1α) in the supernatants of the lung homogenates were measured using commercially available enzyme-linked immunosorbent assay kits (DuoSet^®^ ELISA Development Systems, R&D Systems, Minneapolis, MN, USA) according to the manufacturer’s instructions.

### Statistical Analysis

Data were expressed as M ± SE. The statistical significance of differences among the groups was determined by unpaired *t*-tests or one-way analysis of variance with Dunnett’s multiple range test. Values of *p* < 0.05 were considered to be statistically significant.

## Results

### Diversity and Relative Abundance of Bacteria in PM_2.5_ and PM_10_

In airborne PM_2.5_, the top 20 bacteria species detected using high-throughput sequencing are listed in Table 1. Among them, *Acinetobacter* (26.18%), *Corynebacterium* (21.07%), *Enterococcus* (6.85%), *Aeromonas* (1.58%), and *Pseudomonas* (0.89%) are considered to be potentially harmful to human health. By some contrast, in airborne PM_10_, the top 20 bacteria species detected are listed in Table 1. Among them, *Acinetobacter* (24.84%), *Enterococcus* (20.65%), *Corynebacterium* (7.86%), *Staphylococcus* (1.07%), *Vibrio* (0.06%), and *Neisseria* (0.06%) are considered to be potentially harmful to human health.

**Table 1:** **The diversity and relative abundance of bacteria in air particulate matter (PM_2.__5_ and PM_10_)**.

PM_2.5_				PM_10_			
No.	Name of bacteria	Relative abundance (%)	G+/G−	No.	Name of bacteria	Relative abundance (%)	G+/G−
1	*Acinetobacter*	26.18	G−	1	*Acinetobacter*	24.84	G−
2	*Corynebacterium*	21.07	G+	2	*Enterococcus*	20.65	G+
3	*Psychrobacter*	9.13	G−	3	*Escherichia*	12.54	G−
4	*Enterococcus*	6.85	G+	4	*Bacteroides*	8.89	G−
5	*Lactobacillus*	6.08	G+	5	*Corynebacterium*	7.86	G+
6	*Bacteroides*	5.24	G−	6	*Pseudomonas*	4.38	G−
7	*Faecalibacterium*	3.47	G+	7	*Macrococcus*	3.02	G+
8	*Macrococcus*	3.17	G+	8	*Coprococcus*	2.87	G+
9	*Aeromonas*	1.58	G−	9	*Aeromonas*	2.03	G−
10	*Shewanella*	1.53	G+	10	*Cetobacterium*	1.52	G+
11	*Pseudomonas*	0.89	G−	11	*Staphylococcus*	1.07	G+
12	*Pseudoalteromonas*	0.48	G−	12	*Ruminococcus*	0.85	G+
13	*Cetobacterium*	0.34	G+	13	*Lactobacillus*	0.63	G+
14	*Kocuria*	0.28	G+	14	*Bifidobacterium*	0.28	G+
15	*Halomonas*	0.17	G+	15	*Shewanella*	0.22	G+
16	*Enhydrobacter*	0.10	G−	16	*Faecalibacterium*	0.16	G+
17	*Ruminococcus*	0.08	G+	17	*Kocuria*	0.08	G+
18	*Wautersiella*	0.08	G+	18	*Vibrio*	0.06	G−
19	*Brachybacterium*	0.06	G+	19	*Neisseria*	0.06	G−
20	*Acidiphilium*	0.01	G+	20	*Micrococcus*	0.04	G+

*The top 20 bacteria species in the level of genus and relative abundance were detected in the air particulate matter (PM_2.5_ and PM_10_) using the method of high-throughput sequencing. These bacteria marked in red were known potential pathogenic bacteria to humans*.

*G+, Gram-positive bacteria; G−, Gram-negative bacteria*.

### Diversity and Relative Abundance of Fungi in Airborne PM_2.5_ and PM_10_

In airborne PM_2.5_, the top 20 fungi species were detected using high-throughput sequencing (Table 2). Among them, *Cladosporium* (8.37%), *Aspergillus* (7.18%), *Trichosporon* (4.94%), *Fusarium* (3.84%), and *Cryptococcus* (0.01%) are known potential pathogenic fungi to humans. In airborne PM_10_, the top 20 fungi species detected are listed in Table 2. Among them, *Aspergillus* (6.41%), *Fusarium* (0.08%), *Trichosporon* (0.03%), and *Trichothecium* (0.01%) are considered to be fungi potentially harmful to human health.

**Table 2:** **The diversity and relative abundance of fungi in air particulate matter (PM_2.__5_ and PM_10_)**.

PM_2.5_			PM_10_		
No.	Name of fungi	Relative abundance (%)	No.	Name of fungi	Relative abundance (%)
1	*s-Ascomycota* sp.	29.27	1	*s-Ascomycota* sp.	23.57
2	*Davidiella*	18.34	2	*Davidiella*	16.42
3	*Schizophyllum*	13.41	3	*Schizophyllum*	11.84
4	*Penicillium*	10.58	4	*Alternaria*	9.76
5	*Cladosporium*	8.37	5	*Aspergillus*	6.41
6	*Aspergillus*	7.18	6	*Rhodotorula*	6.37
7	*Trichosporon*	4.94	7	*Tomentella*	4.28
8	*Fusarium*	3.84	8	*Septoria*	2.87
9	*Cladosporium*	1.05	9	*Mortierella*	2.10
10	*s-Trichocomaceae* sp.	0.83	10	*Candida*	1.05
11	*Rhodotorula*	0.74	11	*s-Pleosporaceae* sp.	1.01
12	*s-Agaricomycetes* sp.	0.28	12	*Penicillium*	0.59
13	*s-Sordariomycetes* sp.	0.26	13	*s-Hymenochaetales* sp.	0.31
14	*Tomentella*	0.17	14	*Bjerkandera*	0.14
15	*Suillus*	0.13	15	*Fusarium*	0.08
16	*Candida*	0.06	16	*Cladosporium*	0.06
17	*Hypocrea*	0.06	17	*Peyronellaea*	0.06
18	*Trametes*	0.03	18	*Trichosporon*	0.03
19	*Cryptococcus*	0.01	19	*Trichothecium*	0.01
20	*Guehomyces*	0.01	20	*Selenophoma*	0.01

*The top 20 fungi species in the level of genus and relative abundance were detected in the air particulate matter (PM_2.5_ and PM_10_) using the method of high-throughput sequencing. Based on the technical limitations of high-throughput sequencing method, there are some fungi without identification to the genus level. These fungi marked in red are known potential pathogenic fungi to humans*.

### Endotoxin Levels in PM_2.5_ and PM_10_

During sampling, levels of endotoxin in PM_2.5_ and PM_10_ were detected by LAL assay. Specifically, concentrations of endotoxin in PM_2.5_ were about 28.3 EU mg^−1^, whereas values for PM_10_ were about 31.2 EU mg^−1^.

### Single Stimulation with ${\text{PM}}_{\text{2.5}}^{+},{\text{PM}}_{\text{2.5}}^{-},{\text{PM}}_{\text{10}}^{+},{\text{ and PM}}_{\text{10}}^{-}$

#### Body Weight Gain

No significant differences emerged in the four PM-treated groups compared with the control group (data not shown).

#### Histological Evaluation

**Figure 1:**
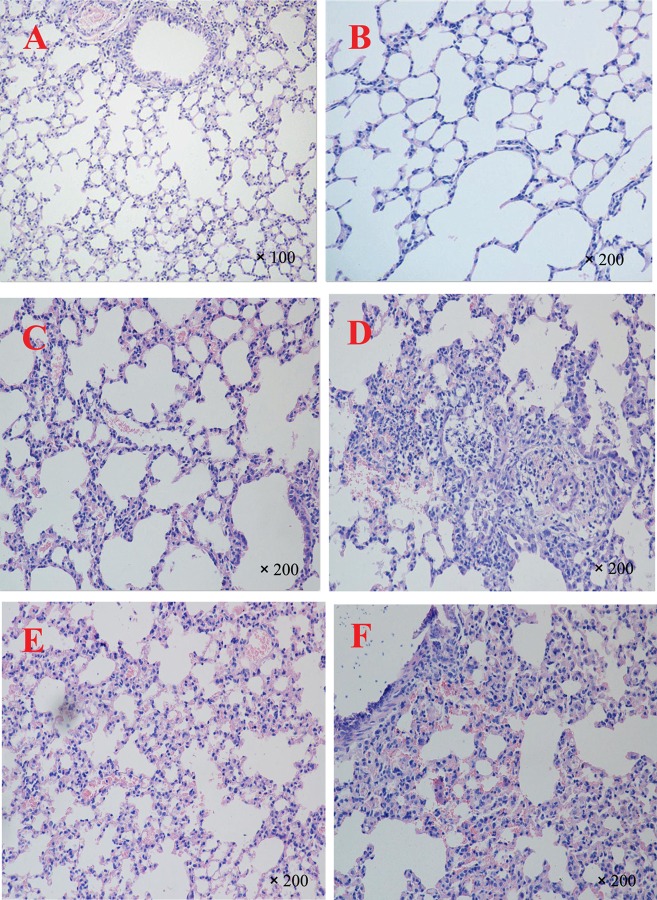
**Representative light microscopy sections of lung tissues of BALB/c mice 72 h after single PM $\left({\text{PM}}_{\text{2.5}}^{+},{\text{PM}}_{\text{2.5}}^{-},{\text{PM}}_{\text{10}}^{+},{\text{ and PM}}_{\text{10}}^{-}\right)$ stimulation**. Normal histopathology can be observed in PBS-treated mice groups **(A,B)**, the lung sections of heat-treated PM_10_ $\left({\text{PM}}_{\text{10}}^{-}\right)$ group **(C)**, the lung sections of non-heat-treated PM_10_ $\left({\text{PM}}_{\text{10}}^{+}\right)$ group **(D)**, the lung sections of heat-treated PM_2.5_ $\left({\text{PM}}_{\text{2.5}}^{-}\right)$ group **(E)**, and the lung sections of non-heat-treated PM_2.5_ $\left({\text{PM}}_{\text{2.5}}^{+}\right)$ group **(F)**. The experiment was performed using six BALB/c mice per group.

As Figure 1C shows, lung sections of Group A exhibited a slight widening of interalveolar septae with fewer neutrophils and pulmonary macrophages than in the control group and were thus scored as 1. Compared with those of Group A, lung sections from Group B (Figure 1D) revealed alveolar distortion with marked infiltration of the pulmonary macrophages and neutrophils into the alveoli and some degree of bleeding and were thus given a score of 2. As Figure 1E shows, lung sections of Group C indicated slightly thickened alveolar walls compared to those of the control group Figures 1A,B, whereas the alveolar walls of Group C exhibited no significant difference compared with those of Group A. However, Group C demonstrated increased infiltration and accumulation of pulmonary macrophages and neutrophils into the alveoli compared with Group A, as well as significant bleeding, and was thus given a score of 2. For Group D (Figure 1F), the degree of lung injury was worse than for Group C. Specifically, alveolar walls were thicker and accompanied by marked infiltration of pulmonary macrophages and neutrophils into the alveoli, pulmonary hemorrhage, and bronchial epithelium with desquamative and squamous metaplastic changes, which merited a score of 3. There was no significant difference in the degree of lung injury between Groups B and D.

#### TLR Expression in the Lungs

**Figure 2:**
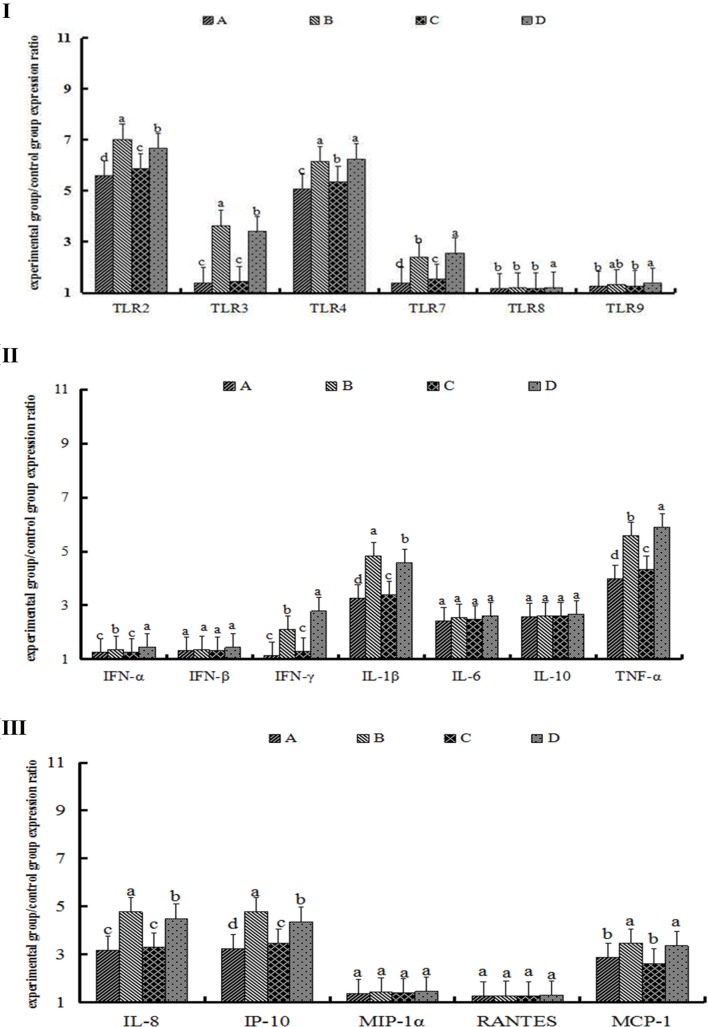
**The expression of toll-like receptors (TLRs) (I), cytokines (II), and chemokines (III) after 72 h of single particulate matter (PM) stimulation**. The protein levels of toll-like receptors (TLRs), cytokines, and chemokines were determined by enzyme-linked immunosorbent assay. **(A)** The heat-treated PM_10_ $\left({\text{PM}}_{\text{10}}^{-}\right)$ group, **(B)** the non-heat-treated PM_10_ $\left({\text{PM}}_{\text{10}}^{+}\right)$ group, **(C)** the heat-treated PM_2.5_ $\left({\text{PM}}_{\text{2.5}}^{-}\right)$ group, and **(D)** the non-heat-treated PM_2.5_ $\left({\text{PM}}_{\text{2.5}}^{+}\right)$ group. Data are presented as means ± SEs. Different letters indicate significant differences (*p* < 0.05) of mean value among the four experimental groups. The experiment was performed using six BALB/c mice per group.

Expression levels of TLR2 in Group A were 5.59-fold compared with those in the control group, whereas concentrations in Group B were 7.02-fold than those of the control group and significantly different from those in Group A (*p* < 0.05). Concentrations of TLR2 in Group C were 5.87-fold than those in the control group and, in Group D, 6.66-fold than those in the control group; the difference between Groups C and D was significant (*p* < 0.05). Moreover, concentrations of TLR2 in Group B were significantly greater than those of Group D (*p* < 0.05, Figure 2I). TLR3, TLR4, and TLR7 showed expression patterns similar to those of TLR2, as results in Figure 2I illustrate. However, the expression concentrations of TLR8 and TLR9 did not show any observable changes (Figure 2I).

#### Cytokine and Chemokine Protein Expression in the Lungs

The expression concentrations of IL-1β in Group A were 3.26-fold than those of the control group, whereas levels of IL-1β in Group B were 4.82-fold than those of Group A, for a statistically significant difference (*p* < 0.05). Levels of IL-1β in Groups C and D were 3.40- and 4.58-fold, respectively, than those of the control group, and the difference between Groups C and D was statistically significant (*p* < 0.05). Furthermore, concentrations of IL-1β in Group B were greater than those in Group D and showed a significant difference (*p* < 0.05) (Figure 2II). IFN-γ and TNF-α showed an expression pattern similar to that of IL-1β; specific results are shown in Figure 2II. Although the expression levels of IFN-α, IFN-β, IL-6, and IL-10 were upregulated compared with those in the control group, there were no significant differences among the PM-treated groups (Figure 2II).

IL-8 protein levels in Groups A and C were 3.17- and 3.31-fold, respectively, but the differences of them were not significant (*p* > 0.05). Expression levels of IL-8 in Group B were 4.76-fold than those of the control group and showed significant differences with levels in Group A (*p* < 0.05). Concentrations of IL-8 in Group D were 4.49-fold and significantly different between Groups C and D (*p* < 0.05). Furthermore, IL-8 protein levels in Group B were greater than those in Group D (*p* < 0.05, Figure 2III). Although IP-10 and MCP-1 showed an expression pattern similar to that of IL-8, expression levels of MIP-1α and RANTES did not show observable changes (Figure 2III).

### Repeated Stimulation with ${\text{PM}}_{\text{2.5}}^{+},{\text{PM}}_{\text{2.5}}^{-},{\text{PM}}_{\text{10}}^{+},{\text{ and PM}}_{\text{10}}^{-}$

#### Body Weight Gain

**Figure 3:**
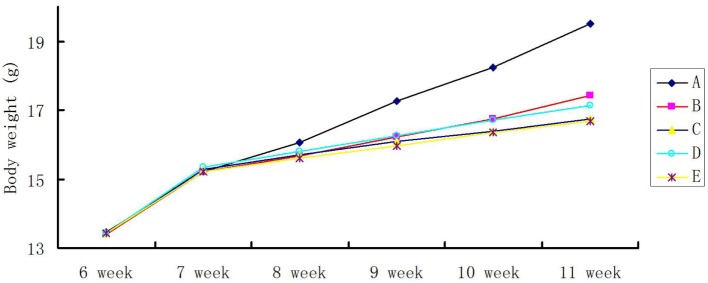
**Time course of body weight gain in repeated particulate matter (PM)-stimulated BALB/c mice**. Body weight was measured twice a week for the entire duration of the experiment. **(A)** PBS-treated group, **(B)** the heat-treated PM_10_ $\left({\text{PM}}_{\text{10}}^{-}\right)$ group, **(C)** the non-heat-treated PM_10_ $\left({\text{PM}}_{\text{10}}^{+}\right)$ group, **(D)** the heat-treated PM_2.5_ $\left({\text{PM}}_{\text{2.5}}^{-}\right)$ group, and **(E)** the non-heat-treated PM_2.5_ $\left({\text{PM}}_{\text{2.5}}^{+}\right)$ group. The experiment was performed using six BALB/c mice per group.

The body weight of mice repeatedly exposed to the four kinds of PM was consistently lower than that of the control group. The average weight of four groups of PM-treated mice in Week 4 of exposure was 17.0 ± 0.5 g, which was significantly lower than that of mice in the control group (19.0 ± 0.5 g; *p* < 0.05; Figure 3A). There were no significant differences among the four groups of PM-treated mice (Figures 3B–E).

#### Histological Evaluation

**Figure 4:**
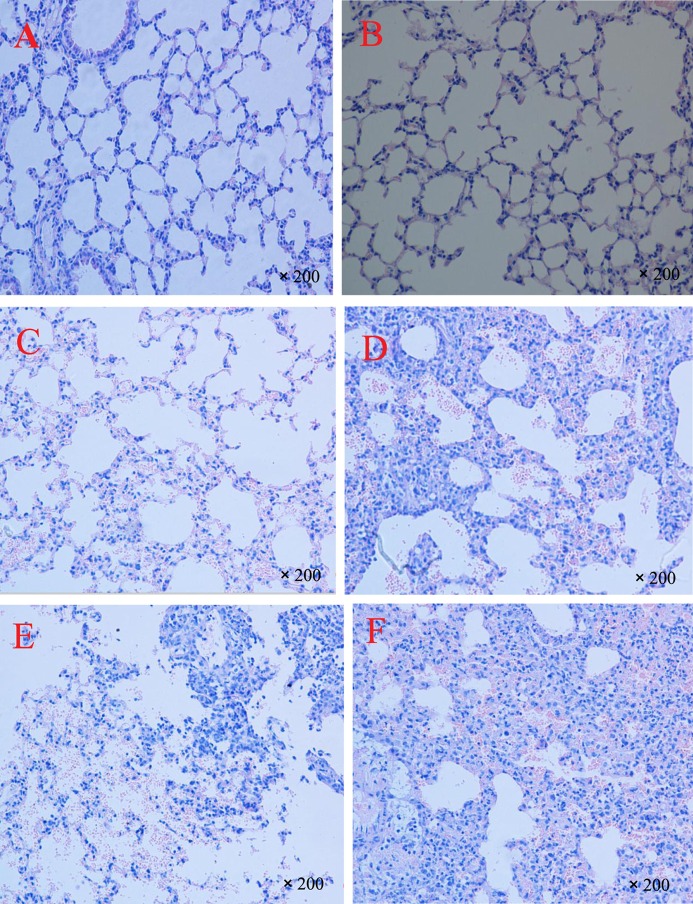
**Representative light microscopy sections of lung tissues of BALB/c mice 72 h after repeated PM $\left({\text{PM}}_{\text{2.5}}^{+},{\text{PM}}_{\text{2.5}}^{-},{\text{PM}}_{\text{10}}^{+},{\text{ and PM}}_{\text{10}}^{-}\right)$ stimulation**. Normal histopathology can be observed in control groups **(A,B)**, the lung sections of heat-treated PM_10_ $\left({\text{PM}}_{\text{10}}^{-}\right)$ group **(C)**, the lung sections of non-heat-treated PM_10_ $\left({\text{PM}}_{\text{10}}^{+}\right)$ group **(D)**, the lung sections of heat-treated PM_2.5_ $\left({\text{PM}}_{\text{2.5}}^{-}\right)$ group **(E)**, and the lung sections of non-heat-treated PM_2.5_ $\left({\text{PM}}_{\text{2.5}}^{+}\right)$ group **(F)**. The experiment was performed using six BALB/c mice per group.

In Group A, part of the alveolar wall structure was incomplete and had minor hyperplasia, and the lung sections demonstrated the exudation of a few inflammatory cells followed by bleeding (Figure 4C) compared to those of the control group (Figures 4A,B); the lungs were thus given a score of 2. As Figure 4D shows, lung sections in Group B showed incomplete alveolar wall structure in the whole field of vision and were markedly thickened, with an increased infiltration and accumulation of neutrophils and pulmonary macrophages. Lung tissue in Group B appeared to be severely damaged compared to tissue in Group A and was scored as 4. In Group C (Figure 4E), mice exhibited serious pulmonary hemorrhage, with a marked infiltration and accumulation of the inflammatory cells, and were given a score of 3. Lung sections of Group D showed severely thickened alveolar walls; most also displayed abnormal alveolar wall structure, with severe pulmonary hemorrhage accompanied by numerous macrophages and neutrophils, and were thus given a score of 4 (Figure 4F).

#### TLR Expression in the Lungs

**Figure 5:**
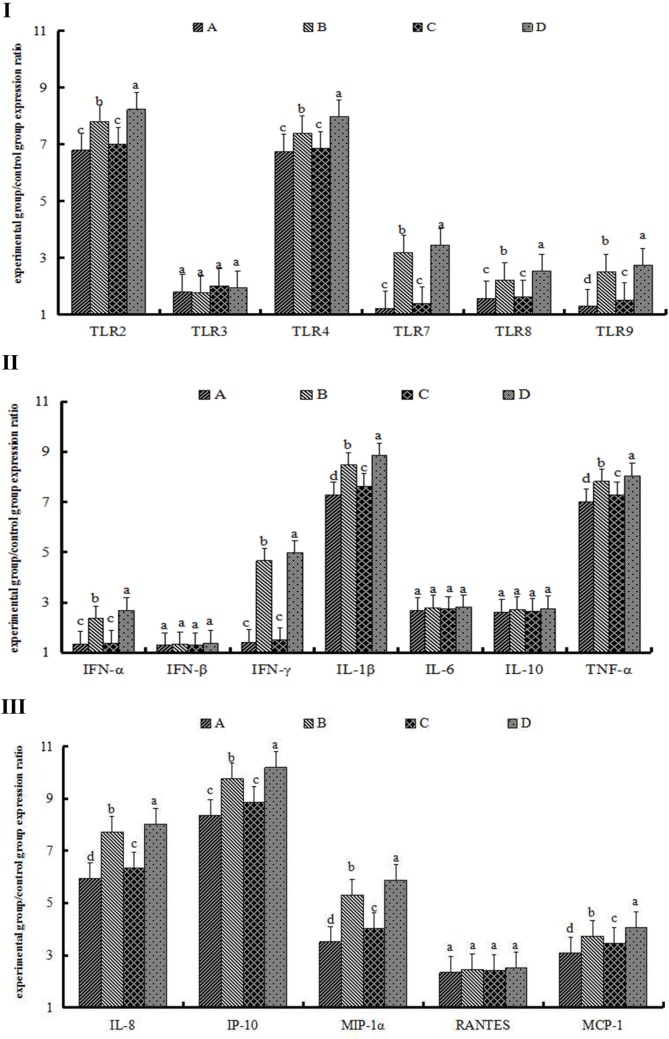
**The expression of toll-like receptors (TLRs) (I), cytokines (II), and chemokines (III) after 72 h of repeated particulate matter (PM) stimulation**. The protein levels of toll-like receptors (TLRs), cytokines, and chemokines were determined by enzyme-linked immunosorbent assay. **(A)** The heat-treated PM_10_ $\left({\text{PM}}_{\text{10}}^{-}\right)$ group. **(B)** The non-heat-treated PM_10_ $\left({\text{PM}}_{\text{10}}^{+}\right)$ group. **(C)** Heat-treated PM_2.5_ $\left({\text{PM}}_{\text{2.5}}^{-}\right)$ group. **(D)** The non-heat-treated PM_2.5_ $\left({\text{PM}}_{\text{2.5}}^{+}\right)$ group. Data are presented as means ± SEs. Different letters indicate significant (*p* < 0.05) differences of mean value among the four experimental groups. The experiment was performed using six BALB/c mice per group.

Expression levels of TLR2 and TLR4 proteins changed significantly, whereas levels of TLR7, TLR8, and TLR9 in Groups B and D were significantly greater than those of the control group (*p* < 0.05). Specific results appear in Figure 5I.

#### Cytokine and Chemokine Protein Expression

Expression levels of IL-1β, TNF-α, IL-8, IFN-γ, and IP-10 proteins increased significantly compared with those in the control group, as similar with single stimulation. However, levels of IL-1β, TNF-α, IL-8, IFN-γ, and IP-10 in the repeated stimulation groups were greater than those in the single stimulation groups (Figures 2II,III and 5II,III). At the same time, the expression level of MIP-1α was significantly upregulated in the repeated stimulation groups and thus different with the single stimulation groups (Figures 2III and 5III).

## Discussion

We investigated whether inhalation challenge with ambient PM_2.5_ and PM_10_ collected from an LBM induced lung inflammation and injury in male BABL/c mice, as well as evaluated differences in the degree of lung injury and inflammatory response with single and repeated PM stimulation. We furthermore determined whether biological components of PM_2.5_ and PM_10_ are essential in mediating observed immune-related inflammatory responses. In the process, the diversity and relative abundance of bacteria and fungi in airborne PM_2.5_ and PM_10_ were investigated and characterized by DNA extraction and sequence analysis of 16S RNA and the internal transcribed spacer region, respectively. The levels of endotoxin in PM_2.5_ and PM_10_ were also detected using LAL assay. Lastly, the biological activation $\left({\text{PM}}_{10}^{+}{\text{ and PM}}_{2.5}^{+}\right)$ and inactivation $\left({\text{PM}}_{10}^{-}{\text{ and PM}}_{2.5}^{-}\right)$ of PM were used to evaluate the lung injury and inflammatory response.

Toll-like receptors play a vital role in inducing and maintaining inflammation by way of pathogen recognition and activating innate immunity in lung tissue (23). Also known to induce adaptive immune response *via* the recognition of various microbial components (24), TLRs can furthermore cause lung injury by eliciting the expression of inflammatory mediators (e.g., specific pro-inflammatory cytokines and chemokines) and by increasing the aggregation of inflammatory cells (25). Each TLR family detects different microbial pathogen-associated molecular pattern and triggers the activation of specific signaling pathways, thereby prompting the transcription of cytokines and chemokines (26). For example, TLR2 primarily recognizes lipoproteins, lipoteichoic acid, and peptidoglycan from Gram-positive bacteria (27). TLR3 engages in recognizing double-stranded RNA, polyriboinosinic–polyribocytidylic acid, and poly (I:C) (28), whereas TLR4 engages in recognizing lipopolysaccharides, the primary component of the outer membrane of Gram-negative bacteria (29). Single-stranded RNA is recognized by TLR7 and TLR8, whereas TLR9 is engaged in recognizing unmethylated bacterial CpG DNA motifs (26).

In the acute phase model, concentrations of TLR2 and TLR4 were significantly increased in all four PM groups. In addition, the expression levels of TLR2 and TLR4 in ${\text{PM}}_{10}^{+}$-treated and ${\text{PM}}_{2.5}^{+}$-treated groups were significantly greater than in the ${\text{PM}}_{10}^{-}$-treated and ${\text{PM}}_{2.5}^{-}$-treated ones (*p* < 0.05). These observations indicate that both TLR2 and TLR4 play a leading role in causing lung injury in mice, which seems to correlate well with previous research on acute lung injury (30, 31).

The role of biological components of PM cannot be overlooked. In previous research on the effects of microbial materials adhered to Asian sand dust, Ichinose et al. (24) indicated that microbial materials adsorbed on the dust can aggravate the degree of lung injury. In our research, endotoxin levels of PM_2.5_ and PM_10_ were also detected using LAL assay. It is well recognized that endotoxin is associated with a constituent of the outer cell wall of Gram-negative bacteria and is a known trigger of airway inflammation and respiratory symptoms. Some studies with animal models suggest that exposure to endotoxin can result in the activation of TLR4 signaling pathways (32), a result that also reflects the importance of biological components of PM. A significant increase in the expression of IL-1β, TNF-α, IL-8, IP-10, and MCP-1 proteins was moreover observed in the acute phase model. In previous research on smoke-induced changes in cytokines and chemokines, Kubo et al. (33) found that a single instance of cigarette smoke exposure can lead to significant increases in the mRNA expression of IL-1β, TNF-α, IL-8, and MCP-1. Taken together, both studies indicate that the expression of IL-1β, TNF-α, IL-8, and MCP-1 can be upregulated in acute lung injury in both mice and guinea pigs. The expression levels of cytokines and chemokines in the ${\text{PM}}_{10}^{+}$-treated and ${\text{PM}}_{2.5}^{+}$-treated groups were significantly greater than in the ${\text{PM}}_{10}^{-}$-treated and ${\text{PM}}_{2.5}^{-}$-treated ones (*p* < 0.05). Furthermore, expression levels of IL-1β, IL-8, IP-10, and MCP-1 in the ${\text{PM}}_{10}^{+}$-treated group were slightly greater than in the ${\text{PM}}_{2.5}^{+}$-treated group. The most likely explanation for that phenomenon is that the biological components are primarily associated with PM_10_ versus fine and ultrafine PM.

Histological examination of acute phase model lung tissues indicated that the degree of lung damage in the four PM-treated groups differed. Specifically, lung injury in the ${\text{PM}}_{10}^{+}$-treated and ${\text{PM}}_{2.5}^{+}$-treated groups was significantly worse than in the ${\text{PM}}_{10}^{-}$-treated and ${\text{PM}}_{2.5}^{-}$-treated ones; the alveolar walls were noticeably thickened, and marked infiltration of pulmonary macrophages and neutrophils into the alveoli were observed compared with the ${\text{PM}}_{10}^{-}$ and ${\text{PM}}_{2.5}^{-}$ groups. The reason for that phenomenon most likely relates to the biological components of PM.

Based on the results of the high-throughput sequencing of bacteria and fungi in PM_2.5_ and PM_10_, several kinds of bacteria and fungi in the top 20 species detected are harmful to human health. For example, *Acinetobacter* can cause respiratory tract infection, septicemia, meningitis, endocarditis, wounds and skin infections, and urinary tract infection, among other conditions (34). The conditional pathogenic bacteria *Enterococcus* and *Pseudomonas* can cause suppurative infection (35), while *Aeromonas* can cause diarrhea, sepsis, and other infections (36). A few species of *Staphylococcus*, especially *Staphylococcus aureus*, can cause suppurative infection and are an important source of cross infection in hospitals (37). Aflatoxin produced by *Aspergillus* can cause seriously toxic and carcinogenic effects upon human health (38), and *Trichosporon* can cause superficial and systemic infection in humans, including hylactic pneumonia (39). Meanwhile, *Cryptococcus neoformans* – a significant emerging fungal pathogen – causes pneumonia and meningitis in humans (40). However, due to the technical limitations of high-throughput sequencing, viruses in airborne PM_2.5_ and PM_10_ were not detected.

In the chronic phase model, a significantly increased expression of TLR2 and TLR4 in the lungs was observed, and those protein concentrations were greater than those of the acute phase model. Consistent with results from the acute phase model, TLR expression levels in ${\text{PM}}_{10}^{+}$-treated and ${\text{PM}}_{2.5}^{+}$-treated groups were greater than in the ${\text{PM}}_{10}^{-}$-treated and ${\text{PM}}_{2.5}^{-}$-treated ones. IL-1β, TNF-α, IL-8, and IP-10 are considered to play an important role in the pathophysiology of lung injury in the chronic phase model followed by IFN-γ, MIP-1, and MCP-1. In addition, the expression of those cytokines and chemokines in the ${\text{PM}}_{10}^{+}$-treated and ${\text{PM}}_{2.5}^{+}$-treated groups were markedly greater than in the ${\text{PM}}_{10}^{-}$-treated and ${\text{PM}}_{2.5}^{-}$-treated ones. The results also suggest that the immunological reactivity between the two sizes of particle seems only to be slightly different; the reason for these results may be due to the fact that some of the top five bacteria in PM_2.5_ and PM_10_ were identical.

In the histological examination of the chronic phase model, significant differences were observed in the degrees of lung damage among the four PM-treated groups. The ${\text{PM}}_{2.5}^{+}$-treated group demonstrated the most severe lung injury, followed by the ${\text{PM}}_{10}^{+}$- and ${\text{PM}}_{2.5}^{-}$-treated groups, respectively. This effect of PM is likely due to the fact that the biological components of PM play a vital role in lung injury in the chronic phase model, or that PM_2.5_ is more efficiently retained in the alveolar lung portion and causes more severe lung injury than coarse fraction PM (i.e., PM_10_) due to its smaller size.

Widespread throughout China, large and small LBMs are often located closer to residential areas. Though arguably more convenient for residents’ day-to-day life, to a certain extent, it also threatens their health. Hosts of chickens, ducks, geese, pigeons, and wild birds sold in LBMs tend to carry high amounts of pathogenic microorganisms, which threaten the health of both customers and employees. In response, China’s government has taken some strict measures, and LBMs in many cities have been shut down or transformed. In general, the more attentive governance of LBMs can benefit public health and the urban environment.

Our results show that short- and long-term exposure to an LBM environment might harm people’s health, especially that of their lungs. At the same time, the effects of microorganisms found in PM that cause pulmonary damage cannot be overlooked. Altogether, the results of our study should help to spread awareness of LBM environments and how they impact the health of employees working there, as well as their customers.

## Author Contributions

KM and TC designed experiments; KM and BW carried out experiments; JG, YC, and MY analyzed the experimental results and developed analysis tools; and KM and LW wrote the manuscript.

## Conflict of Interest Statement

The authors declare that the research was conducted in the absence of any commercial or financial relationships that could be construed as a potential conflict of interest.
